# Tripeptide Arg-Gly-Asp (RGD) modifies the molecular mechanical properties of the non-muscle myosin IIA in human bone marrow-derived myofibroblasts seeded in a collagen scaffold

**DOI:** 10.1371/journal.pone.0222683

**Published:** 2019-10-01

**Authors:** Yves Lecarpentier, Vincent Kindler, Marie-Luce Bochaton-Piallat, Antonija Sakic, Victor Claes, Jean-Louis Hébert, Alexandre Vallée, Olivier Schussler

**Affiliations:** 1 Centre de Recherche Clinique, Grand Hôpital de l’Est Francilien, Meaux, France; 2 Department of Specialties in Medicine, Hematology Service, Geneva University Hospital, Switzerland Faculty of Medicine, Geneva, Switzerland; 3 Department of Pathology and Immunology, Centre Médical Universitaire Geneva, Faculty of Medicine, Geneva, Switzerland; 4 Department of Pharmaceutical Sciences, University of Antwerp, Wilrijk, Belgium; 5 Institut de Cardiologie, Hôpital de la Pitié-Salpétrière, Paris, France; 6 Paris-Descartes University, Diagnosis and Therapeutic Center, Hypertension and Cardiovascular Prevention Unit, Hôtel-Dieu Hospital, Paris, France; 7 DRCI (Délégation à la Recherche Clinique et Industrielle) Hôpital Foch, Suresnes, France; 8 Department of Cardiovascular Surgery, Research Laboratory, Geneva University Hospitals, Geneva, Switzerland; Touro College, UNITED STATES

## Abstract

Mesenchymal stem cells (MSCs) were obtained from human bone marrow and amplified in cultures supplemented with human platelet lysate in order to generate myofibroblasts. When MSCs were seeded in solid collagen scaffolds, they differentiated into myofibroblasts that were observed to strongly bind to the substrate, forming a 3D cell scaffold network that developed tension and shortening after KCl stimulation. Moreover, MSC-laden scaffolds recapitulated the Frank-Starling mechanism so that active tension increased in response to increases in the initial length of the contractile system. This constituted a bioengineering tissue that exhibited the contractile properties observed in both striated and smooth muscles. By using the A. F. Huxley formalism, we determined the myosin crossbridge (CB) kinetics of attachment (f1) and detachment (g1 and g2), maximum myosin ATPase activity, molar myosin concentration, unitary CB force and maximum CB efficiency. CB kinetics were dramatically slow, characterizing the non-muscle myosin type IIA (NMMIIA) present in myofibroblasts. When MSCs were seeded in solid collagen scaffolds functionalized with Arg-Gly-Asp (RGD), contractility increased and CB kinetics were modified, whereas the unitary NMMIIA-CB force and maximum CB efficiency did not change. In conclusion, we provided a non-muscle bioengineering tissue whose molecular mechanical characteristics of NMMIIA were very close to those of a non-muscle contractile tissue such as the human placenta.

## Introduction

Recently, we reported that mesenchymal stem cells (MSCs) derived from human bone marrow (BM) and seeded in solid 3D-collagen scaffolds differentiated into myofibroblasts in the presence of human platelet lysate (HPL) and contracted when exposed to KCl or an electrical field [1]. Myofibroblasts in collagen scaffolds were shown to contain α-smooth muscle actin (α-SMA) and non-muscle myosin II (NMMIIA), which were colocalized, but no muscle myosin. Myofibroblasts are non-muscle contractile cells [2] that encourage wound healing by secreting collagen and inducing wound contraction. The active retraction of contractile myofibroblasts in granulation tissues allows wound contraction which promotes tissue reconstruction [3]. Myofibroblasts also reside in normal non-inflammatory tissues such as the human placenta [4], as well as in several pathological cancer-associated stroma where they may result in tumor growth and fibrotic processes [5, 6, 7]. After amplification, MSCs can differentiate *in vitro* into many cell types, including fibroblasts and myofibroblasts [8]. In 2D culture, the differentiation of fibroblasts into myofibroblasts is encouraged by several factors such as the transforming growth factor- β (TGF-β) [9, 10]. This is present in high concentrations in HPL routinely used to amplify MSCs [11]. The presence of collagen and fibronectin and a certain degree of stiffness of the environment where cells reside, also impact the differentiation of MSCs towards myofibroblasts [12, 13].

Earlier studies have assessed the *in vitro* contractile properties of the human placenta [14]. This non-muscle tissue harbors a high density of myofibroblasts [4], which are located in stem villi and present mechanical properties similar to those observed in all striated and smooth muscles: 1°) they are activated by either an electrical field or KCl exposure; 2°) they relax after myosin crossbridge (CB) inhibition or by decreasing the intracellular Ca^2+^ concentration; 3°) they conform to the Frank-Starling mechanism [15, 16]: 4°) they present a hyperbolic relationship between peak shortening velocity (V) and the level of isotonic tension (T) [17]. Such a hyperbolic T-V relationship makes it possible to apply the A. Huxley formalism [18] and, in turn, to compute the kinetics of attachment and detachment of myosin CBs, maximum myosin ATPase activity, unitary CB force, maximum CB efficiency and myosin molar concentration [19]. The molecular motor of myofibroblasts is the NMMIIA [20, 21]. NMMIIA, whose kinetics are considerably slower than those of type II muscle myosin (MMII) [19, 22], has been found to be the molecular motor driving the contraction in both human placenta [20] and MSC-seeded collagen scaffolds [1].

The solid 3D collagen scaffold were functionalized with the linear Arg-Gly-Asp (RGD) before MSC-seeding. The covalent binding of the linear RGD motif to same solid collagen scaffolds has been shown to improve the contractility of rat cardiomyocytes seeded in such structure [23]. In this study, we showed that the Frank-Starling phenomenon and the hyperbolic T-V relationship were observed in MSC-seeded collagen scaffolds. Moreover, we assessed the molecular contractile properties of MSCs-derived myofibroblasts in terms of the kinetics of attachment and detachment of NMMIIA- CBs, maximum NMMIIA- ATPase activity, unitary CB force, maximum CB efficiency and NMMIIA molar concentration, and we investigated whether the functionalization of solid collagen scaffold with the RGD motif impacted these molecular parameters.

## Materials and methods

### Background

This study was approved by the local ethics committee of Geneva University Hospital, named « Commission Cantonale Ethique de la Recherche Scientifique de Genève » (CCER). The human femoral heads were collected during surgical interventions for hip replacements, according to the CCER, and after patients were informed and gave their written consent. MSC extraction, amplification and MSC seeding in 3D-solid collagen scaffolds have been previously described in detail [1]. Thus, BM-derived MSCs seeded in collagen scaffolds have been shown to form an adherent cell stroma with well-organized microfilaments expressing α-SMA and a high level of NMMIIA, but they are devoid of muscle myosin. Experiments conducted in the present study have been performed within the same period and with the same biological samples used in our previous study [1]. Thus, we have performed the present experiments with MSCs obtained from the same donors, loaded on collagen foam scaffolds which have been used for the experiments described in [1]. These cells were positive for α-smooth muscle actin and non-muscle myosin type IIA (NMMIIA) characterizing myofibroblasts [2, 4, 20]. They were also found to be positive for CD44, CD54, CD73, CD90, CD105, CD140b, CD146, and negative for HLA-DR, CD31, CD45, and CD56, which is a phenotype compatible with both myofibroblasts and MSCs. Cell counting was made by means of a hemocytometer as previously described [1].

### Covalent binding of the RGD motif to the solid 3D collagen scaffolds

Scaffolds are a solid highly porous collagen structures obtained by physical reticulation by dehydrothermal (DHT) polymerisation of bovine dermal collagen type I and type III. Scaffolds are commercially available for clinical implantation as hemostatic sponge. The 3D structure of collagen RGD scaffolds was obtained by electron microscopy showing a classical ultrastructure of sponge [23]. The functionalization of the solid clinical hemostatic sponge (Avitene^™^ Ultrafoam collagen (Bard Limited, Crawley, UK, ref 1050050) with the linear RGD motive of fibronectin has been reported [23]. Structural properties of solid collagen scaffolds were as follows: pore size between 20–200 μm, interconnectivity 100%, anisotropy 1.32±0.14, total porosity 94%. The high porosity was very important for the chemical functionalization of the scaffold but also for cellular migration and nutriment diffusion.

The full linear RGD peptide of fibronectin, i.e., glycine-arginine- glycine-aspartic acid-serine peptide (GRGDS; G4391, Sigma Aldrich, Lyon, France) was used. Briefly, for functionalization, the RGD motif needs to be bound to the scaffold. The use of a spacer arm between 30–40 A is optimal for the RGD presentation and favorable for integrin-receptor clustering and activation [24, 25]. The conjugation occurred between NH2 sites of the RGD motif and NH2 sites of the collagen by using the water soluble heterobifunctional crosslinker Sulfosuccinimidyl 6-[3-(2-pyridyldithio)-propionamido] hexanoate, (Sulfo-LC-SPDP; Pierce Biochemical, Rockford, IL). The RGD motif to the collagen scaffold occurred in a dose-dependent manner. We and others have reported the optimum time and concentration using Sulfo-LC-SPDP with collagen in solution [26] or in the form of a solid 3D scaffold [23]. Sulfo-LC-SPDP was used to functionalize the solid DHT collagen with the RGD peptide and allowed also the introduction of a spacer arm of 36 Å between the collagen NH2 sites and the NH2 sites of the RGD peptide. This distance has been shown to be optimal for integrin-receptor clustering and activation [24, 25]. The last step of fixation was accompanied by the release of pyridine—2-thione into solution that was monitored by absorbance at 343 nm and correlated to the fixation of the RGD to the collagen. GRGDS are covalently bound to collagen sponges by using the crosslinker. The coupling implied three successive chemical steps: The crosslinker reacts with the NH2 sites present on the collagen scaffold during 48 h (i.e., 50 μl of Sulfo-LC-SPDP) for 10 mg of collagen). Excess reagent was removed by washing the matrix in PBS. Free sulfhydryl groups were reduced with dithiothreitol during 1 h. Excess dithiothreitol was removed by washing. A pyridyldithio group was added separately to the NH2 site on GRGDS by incubating GRGDS with Sulfo-LC-SPDP in 1:2 molar ratio, at room temperature during 48 h in PBS. Reduced SPDP-collagen scaffold was mixed with pyridyldithio-GRGDS peptides during 48 h in PBS. In the last step of the binding, the reduced SPDP-collagen scaffold was mixed with pyridyldithio-GRGDS peptide during 48 h in PBS. This last step was monitored spectrophotometrically by quantifying the release of pyridine-2-thione into solution by absorbance at 343 nm and extinction coefficient for pyridine-2-thione at 343 nm (i.e., 8080 M^-1^. cm^-1^). The level of SPDP-modification was monitored by the release of pyridine-2-thione in the medium. Moles of SPDP per mol of protein (GRGDS) is equal to the absorbance change at 343nm divided by 8080 and multiplied by the molecular weigth of GRGDS and divided by mg/ml of GRGDS. With this protocol for 10 mg of collagen, we achieved a level of peptide functionalization of 70±4%; n = 7). MSCs were used after 4 passages and 10^5^ MSCs were seeded in each scaffold. The time during which MSCs were cultured in scaffolds was 3 to 4 weeks. Some preliminary experiments were done after 8 days of culture and this led to the same level of MSC-seeded scaffold contractility.

### Experimental mechanical set-up for scaffolds

**Fig 1:**
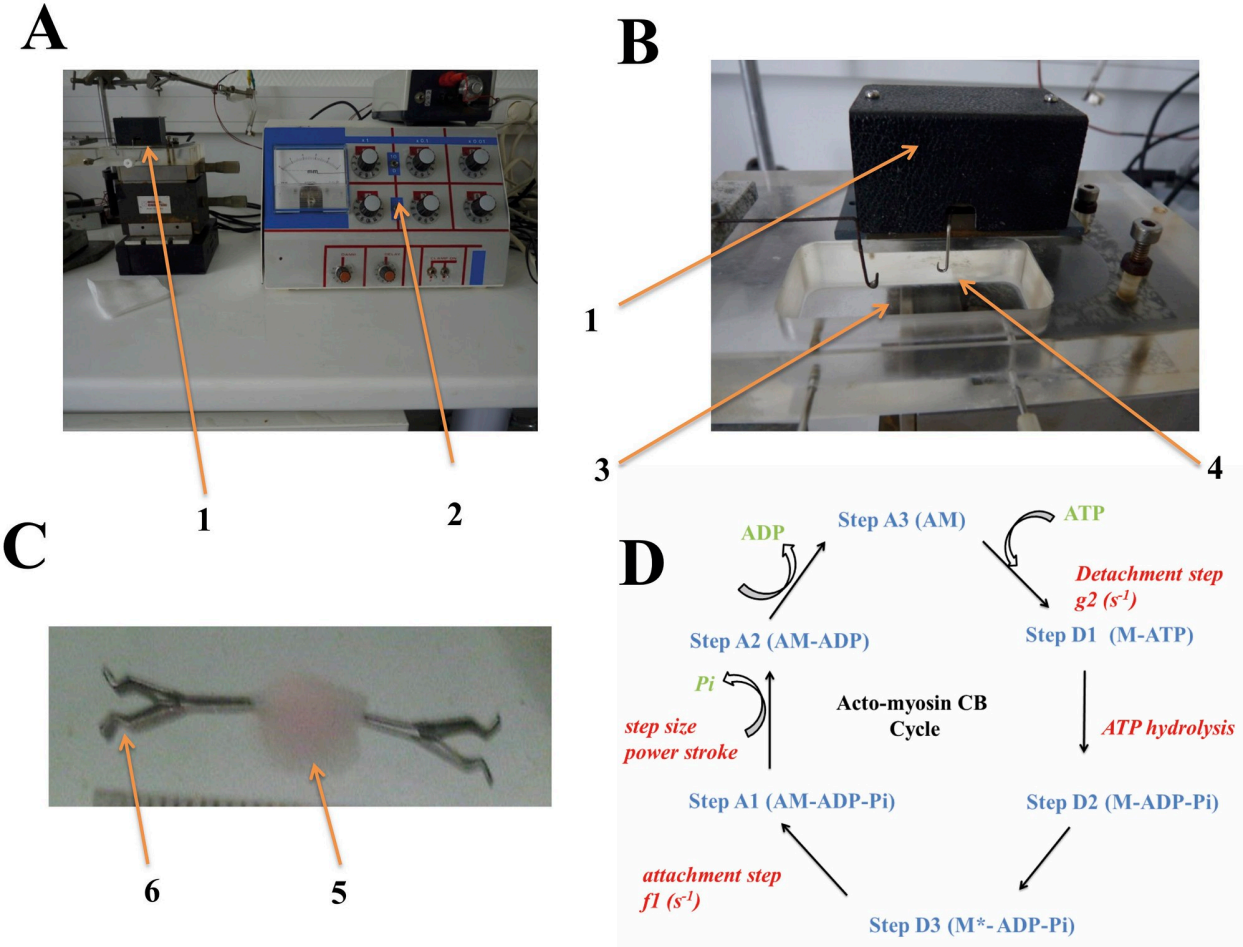
**Panel A**: overview of the experimental set-up, with the force and length transducer (1) and the electromagnetic system (2); **Panel B**: force and length transducer (1); the bath (3) in which the collagen scaffold is immersed; the moving arm lever of the transducer (4); **Panel C**: the collagen scaffold (5) attached by 2 clips (6) that are connected to the moving arm lever of the transducer (4) and a fixed hook; **Panel D**: Myofibroblast NMMIIA-CB cycle. The CB cycle is composed of six different conformational steps, i.e., three detached steps (D1, D2, and D3) and three attached steps (A1, A2, and A3). The myosin molecular motor is the non-muscle myosin type IIA (NMMIIA). Transition A**3 →** D1 is the ATP binding step that induces CB detachment after ATP binding with the actin (A)-myosin (M) complex (AM). The rate constant for detachment is g_2_. AM + ATP **→**A + M-ATP. Transition D1 **→** D2 is the ATP hydrolysis: M + ATP **→** M-ADP-Pi. Transition D2 **→** D3 is M-ADP-Pi **→** M*-ADP-Pi. D3 is the step with the highest probability. Transition D3 **→** A1 is the attachment state: the myosin head (M*-ADP-Pi) binds with A and the rate constant for attachment is f_1_: M*-ADP-Pi + A **→** AM-ADP-Pi. Transition A1 **→**A2 is the power stroke which is triggered by the Pi release: AM-ADP-Pi **→** AM-ADP + Pi. The power stroke is characterized by the generation of a unitary CB force and the CB step. Transition A2 **→** A3 is the release of ADP: AM-ADP **→** AM + ADP. NMMIIA contains three pairs of chains and two systems regulate its activity, i.e., the calcium-calmoduline-myosin light chain kinase (MLCK) and the Rho/ROCK/myosin light chain phosphatase [12, 45, 46]. NMMIIA molecules assemble into bipolar filaments, allowing myosin sliding along actin in an anti-parallel manner. The power stroke occurs with a tilt of the NMMIIA head and produces a force of a few pico Newtons and a displacement of a few nanometers. Importantly, the kinetics of NMMIIA are extremely slow [19, 22].

BM-derived MSC-laden scaffolds, both with and without the RGD motif, were used to test their contractile properties. Each scaffold was mounted in an experimental chamber containing a Krebs-Henseleit solution, bubbled with 95% O2–5% CO2 to insure a pH of 7.4. Experiments were carried out at room temperature, and at resting length (Lo), i.e., with a resting tone (RT) that induced neither spontaneous shortening nor spontaneous lengthening of the scaffold. At the end of the experiment, the cross-sectional area (CSA) of the scaffold was calculated (in mm^2^) from the weight/Lo ratio. Scaffolds were chemically stimulated by KCl (0.05M). The electromagnetic force-length transducer has been previously described [14] (Fig 1A–1C).

### Passive mechanical parameter of scaffolds: Young’s modulus E

**Fig 2:**
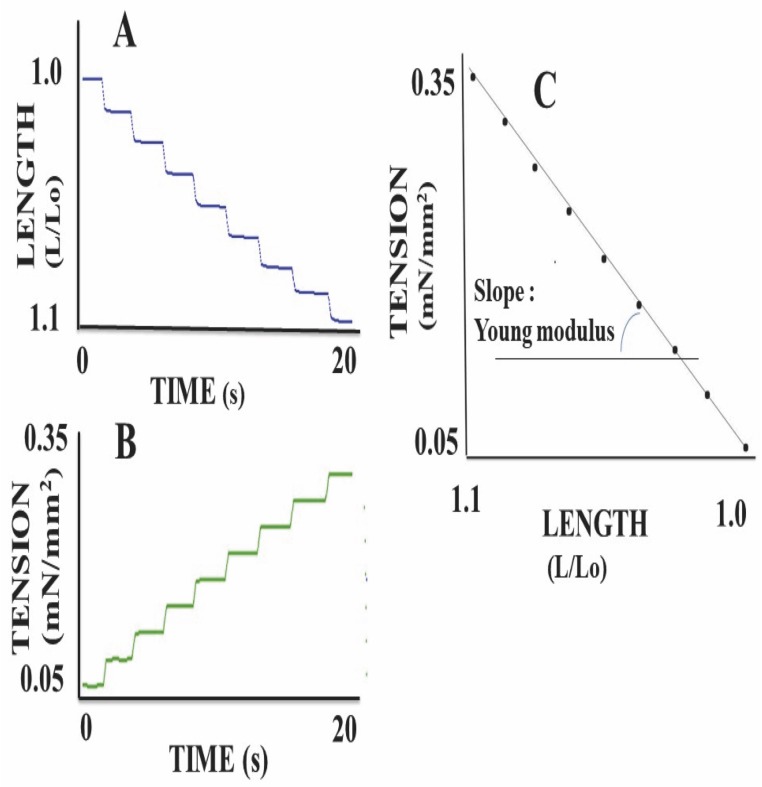
Determination of the Young modulus. Before KCl stimulation, successive load clamps (**panel B**) were imposed on the scaffold. This induced a progressive elongation of the scaffold (**panel A**). **In panel C**, tension is shown as a function of scaffold length. The slope of this relationship provides the Young modulus.

Mechanical measurements were made after a 30-minute period of stabilization. The Young modulus E was determined before stimulation of the scaffold by KCl. The stress σ (in Pascal; Pa) was the force F (in N) per CSA (in m^2^) imposed on the scaffold and σ = F/CSA (Fig 2B). The strain ε (in m/m) represented the scaffold deformation due to the force F imposed on it (Fig 2A). The strain ε is the change in length (dL) divided by its original value (L in m) and ε = dL/L. The Young modulus E = stress / strain = σ / ε = (F/CSA) / (dL/L). Successive increments in tension were imposed on the scaffold by means of 0.1 mN load-clamps. This generated a progressive scaffold elongation. The slope of the stress-strain relationship represented the Young modulus (Fig 2C).

### Active mechanical parameters of MSC scaffolds

**Fig 3:**
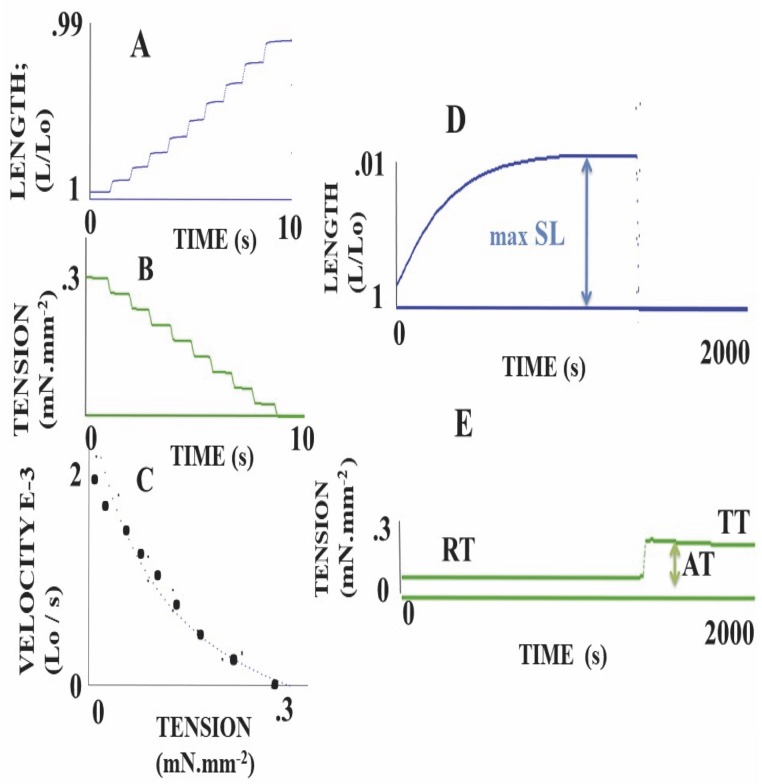
Mechanical parameters of KCl-activated scaffolds. **Panels A, B and C**: Determination of the T-V hyperbolic relationship. In a KCl-activated scaffold, tension (**panel B**) was progressively decreased by successive load clamps. Just after a short transient overshoot, induced by the load clamp, the peak velocity corresponding to a new isotonic load level was measured. **Panel C** represents the hyperbolic T-V relationship. **In panel D** (length) and E (tension), the addition of 0.05 M KCl in the bath immediately induced a shortening of the scaffold. When the shortening length (max SL) reached a plateau, the isometric condition was imposed, and the tension immediately reached total tension (TT). Active tension (AT) = TT-AT.

Scaffolds were preloaded at RT. KCl (0.05M) was then introduced in the experimental chamber. This induced an immediate isotonic shortening of the scaffold (Fig 3D). A plateau was reached after approximately 1000 s, determining the maximum amplitude of shortening length (max SL). When peak shortening was reached, isometric conditions were quickly imposed on the scaffold, causing its length to immediately return to Lo (Fig 3D and 3E). The scaffold then developed an active isometric tension (AT) equal to total isometric tension (TT) minus RT.

### The Frank-Starling mechanism

**Fig 4:**
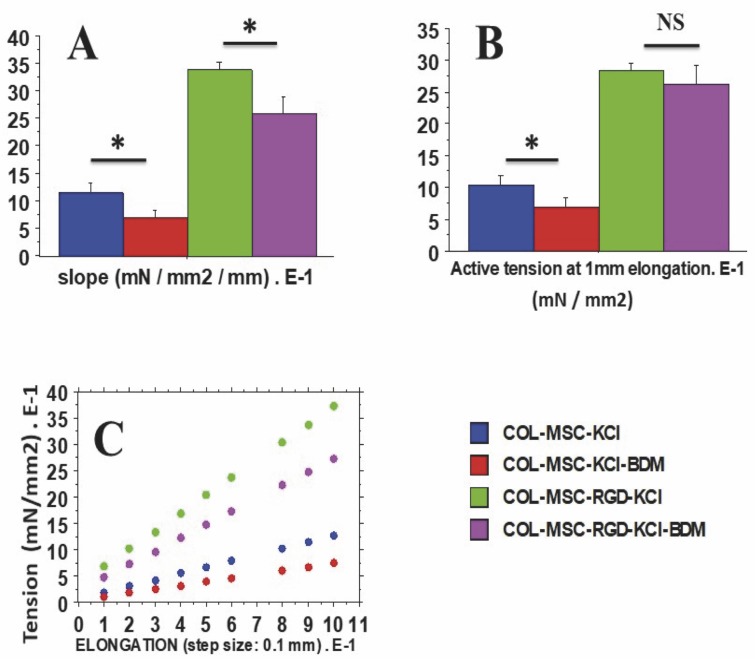
Frank-Starling phenomenon. Total isometric tension (T in mN) was measured as a function of increasing initial length (Li in mm) of scaffolds (T-Li relationship), with and without RGD. This curve was first drawn in KCl-activated scaffolds, then in KCl-activated scaffolds + BDM. **Panel A**: slope of the T-Li relationship; **Panel B**: Active tension after 1 mm elongation of the scaffold; **Panel C**: T-Li relationship drawn after successive length clamps of 0.1 mm.

The Frank-Starling mechanism [15, 16] was highlighted when the active isometric tension (AT = TT- RT) increased with the preload that determined the initial length of the contractile system. The Frank-Starling relationship characterizes the contractile level of the system and is measured by the isometric active tension observed with increasing the initial length. The slope of the isometric tension-length relationship determined the level of contractility (Fig 4A). Isometric tension was measured at 1 mm elongation (Fig 4B). The isometric tension-elongation relationships were determined according to the elongation of the scaffold (Fig 4C). These relationships were successively measured after exposure to KCl (0.05M) and then after the further addition of 2,3-butanedione monoxime (BDM) to KCl. BDM is an inhibitor of NMMII-CBs and induces a decrease in contractility, i. e. a negative inotropic effect. Thus, the Frank-Starling curves were measured at 2 different levels of contractility: under the influence of KCl alone, then that of KCl plus BDM (Fig 4A, 4B and 4C).

### The Hill hyperbolic T-V relationship

The A.V. Hill hyperbolic tension-velocity (T-V) relationship [17] was determined by imposing successive decreases in load (T) from total isometric tension (TT) until zero-load, and under 0.05 M KCl in the bath [14] (Fig 3A, 3B and 3C). The T-V relationship was fitted according to the A.V. Hill equation (T + a) (V + b) = [TT + a] b, where -a and -b were the asymptotes of the hyperbola. For numerous muscle and non-muscle tissues, the T-V relationship was accurately fitted by means of a hyperbola. Maximum isometric tension, i.e. maximum total force normalized per cross-sectional area (TT, in mN.mm^−2^) was measured from fully isometric conditions. Vmax was the maximum shortening velocity at zero load. The G curvature of the T-V relationship was TT / a = Vmax / b [17]. V was the maximum isotonic velocity corresponding to the isotonic tension (T) level.

### The A. Huxley phenomenological formalism

The A. Huxley formalism [18] enables the computation of numerous indices characterizing the molecular behavior of NMMIIA-CBs and the kinetics of the different steps of the CB cycle (Fig 1D). For this formalism to be used, the contractile system must exhibit a hyperbolic T-V relationship. Thus, both asymptotes -a and -b and the G curvature of the T-V relationship are part of the Huxley equations. The rate of total energy release (EHux) and isotonic tension (PHux) are a function of the maximum shortening velocity (V) of the contractile structure according to the following equations:

$$E_{Hux}=(Ne)(\frac{h}{2\ell })(\frac{f_{1}}{f_{1}+g_{1}})[g_{1}+f_{1}(\frac{V}{\phi })[(1-\text{exp}\left(-\phi \right)]$$

$$P_{Hux}={N(\frac{sw}{2\ell })(f_{1}+g_{1})[1-(\frac{V}{\phi })[1-(\frac{V}{\phi })[1-\text{exp}\left(-\frac{\phi }{V}\right))(1+(\frac{1}{2})(\frac{f_{1}+g_{1}}{g_{2}})}^{2}\left(\frac{V}{\phi }]\right]$$

f1 was the maximum value of the rate constant for CB attachment; g_1_ and g_2_ were the maximum values of the rate constants for CB detachment; w was the maximum mechanical work of a unitary CB (w / e = 0.75) and e was the free energy required to split one ATP molecule. The standard free energy ΔG°’_ATP_ was—60 kJ /mol. The value used for e was 10 ^−19^ J [27]. The tilt of the CB head relative to actin varied from 0 to h; f_1_ and g_1_ corresponded to a tilt from 0 to h and g_2_ corresponded to a tilt > h; Φ = (f_1_ + g_1_) h / 2 = b; N was the number of cycling CBs per mm^2^ at peak isometric tension. The molecular step size h represented the translocation distance of the actin filament per ATP hydrolysis, produced by the tilt of the myosin head. Parameter *ℓ* represented the distance between two successive actin sites with which any myosin site can combine. According to the A. Huxley conditions (*ℓ* >>h), the h and *ℓ* values were h = 10 nm and *ℓ* = 28.6 nm, which is close to the semi-helicoidal turn of the actin filament [28]. The value of h was confirmed by the three-dimensional head structure of the muscle myosin II [29, 30]. Values of f_1_, g_1_, and g_2_ were obtained from the following equations [31]:

$$G=f_{1}/g_{1}$$

$$g_{1}=2wb/ehG$$

$$g_{2}=2Vmax/h$$

$$\pi o=(\frac{w}{l})\times \left[\frac{f_{1}}{f_{1}+g_{1}}\right]$$

$$kcat=(\frac{h}{2l})\times \left[\frac{f_{1}g_{1}}{f_{1}+g_{1}}\right]$$

πo was the unitary CB force. NMMIIA content was calculated from the CB number per g of tissue (nM.g^−1^) and the Avogadro number. The maximum NMMIIA-ATPase activity was the product of the catalytic constant (kcat, in s^-1^) and NMMIIA content. The rate of mechanical work (W_M_) was equal to the product PHux x V [32]. At any given load level, the CB efficiency of the contractile tissue was defined as the ratio of W_M_ and EHux. Maximum efficiency (Eff.max) was the maximum value of CB efficiency.

### Statistics

Data were expressed as means ± standard deviation (SD). Student’s unpaired t-test was used for comparisons of parameters between collagen scaffolds seeded with MSCs and functionalized or not, with RGD. On Figures, *: p < 0.05; * *: p < 0.01; * * *: p < 0.001. NS: non significant. A p value < 0.05 was considered statistically significant. Linear regressions were performed by means of the least squares method.

## Results

**Fig 5:**
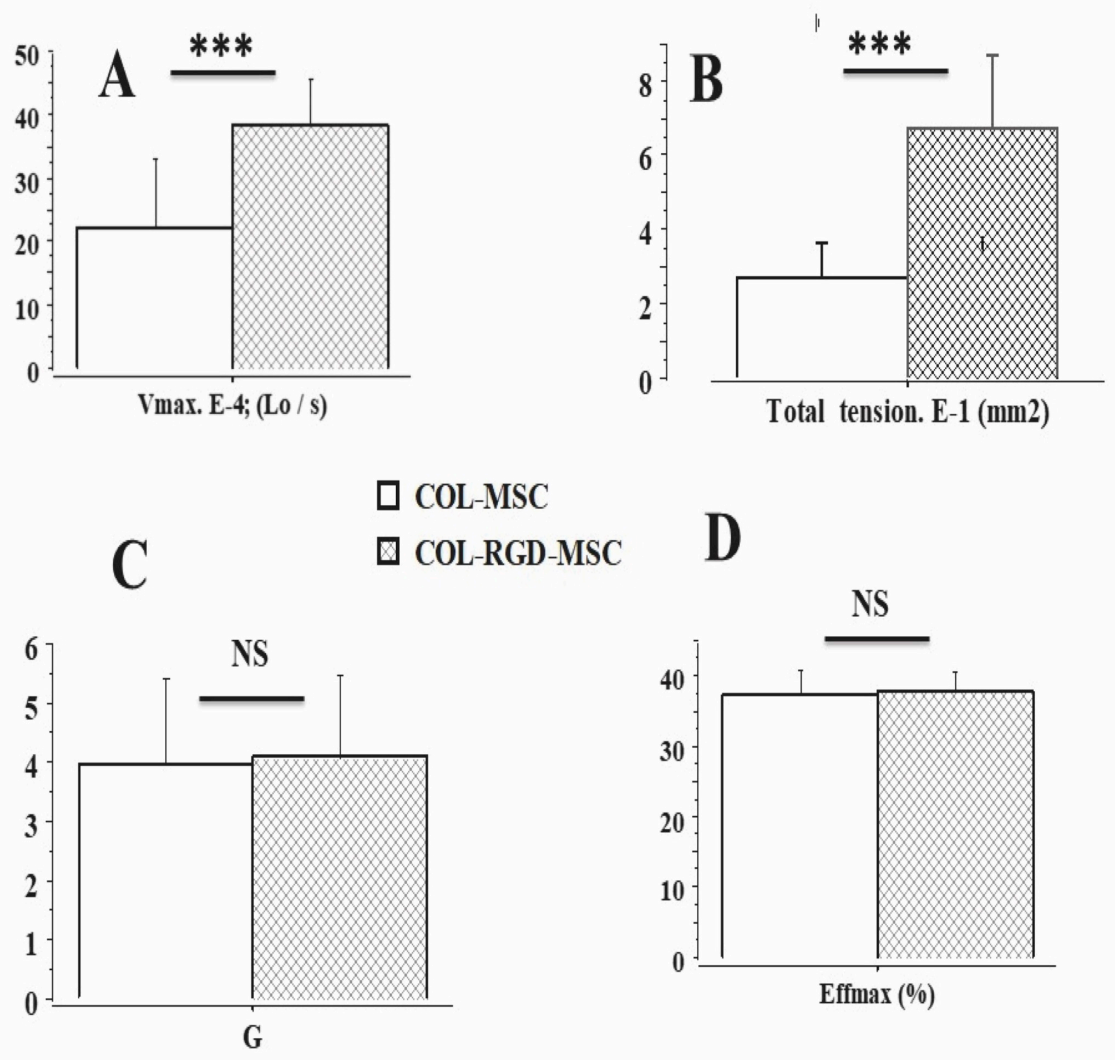
Mechanical parameters of scaffolds± SD. **Panel A**: maximum unloaded shortening velocity; **Panel B**: Total isometric tension; **Panel C**: Curvature of the T-V relationship; **Panel D**: maximum CB efficiency (Eff max).

**Fig 6:**
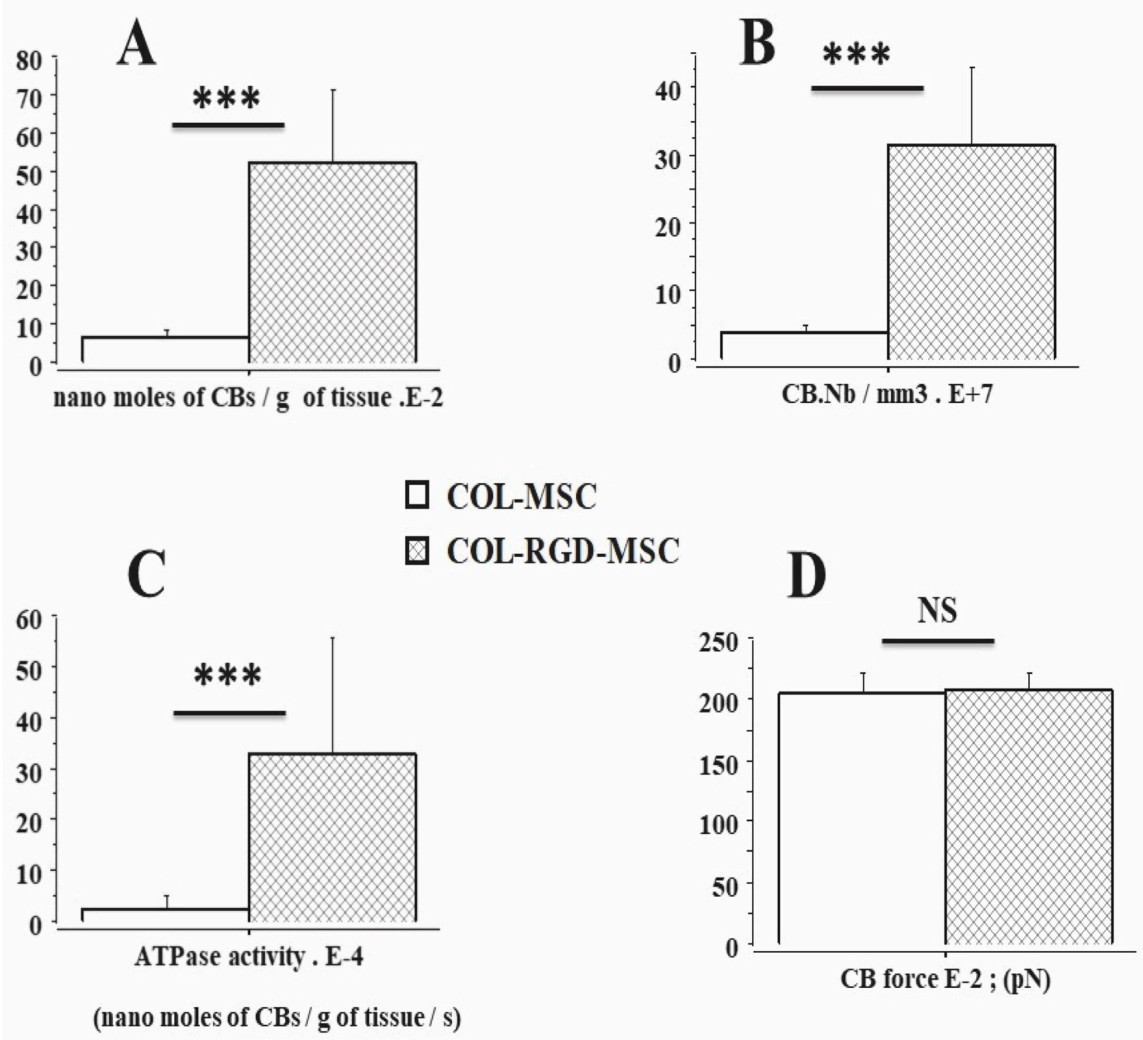
Molecular mechanical parameters of the NMMIIA in COL-MSC and COL-RGD-MSC scaffolds. **Panel A**: Nanomolar concentration of myosin; **Panel B**; Number of myosin CBs per mm^3^; **Panel C**: NMMIIA-ATPase activity; **Panel D**: NMMIIA unitary CB force.

**Fig 7:**
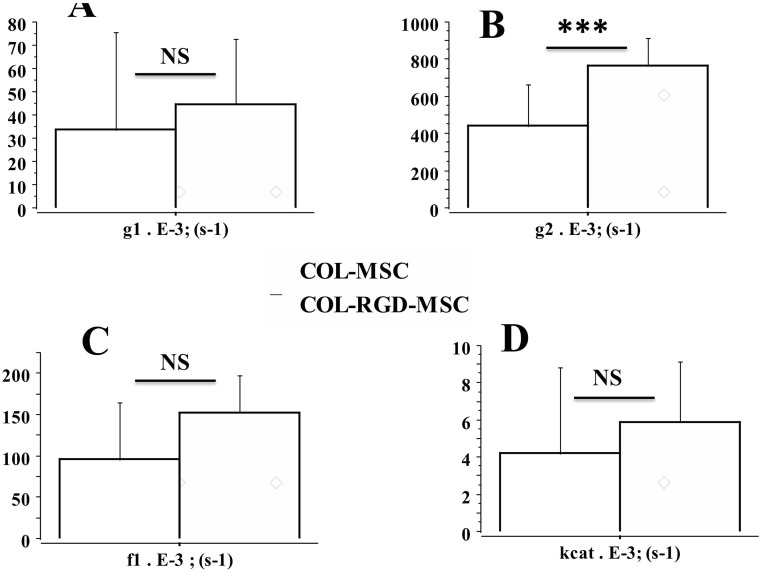
Molecular kinetics of the NMMIIA in COL-MSC and COL-RGD-MSC scaffolds. **Panel A**: g1: CB detachment constant; **Panel B**: g2: CB detachment constant; **Panel C**: f1: CB attachment constant; **Panel D:** kcat: catalytic constant.

The Results section was subdivided into 5 subsections. In the subsections 1 and 2, physical properties of collagen scaffolds seeded with MSCs, with or without RDG (subsection 1: Physical properties of scaffolds seeded with MSCs), and their passive mechanical properties (subsection 2: Passive parameter: The Young modulus of scaffolds seeded with MSCs) (Fig 2) were presented. In these two subsections, the collagen scaffolds were studied with or without RDG and before any chemical stimulation by KCl. The presence of RGD did not modify the Young modulus. In subsections 3, 4 and 5 of the Results section, the active mechanical properties of collagen scaffolds with or without RDG were studied under chemical stimulation by KCl. Subsection 3 reported their classical mechanical properties (maximum unloaded velocity (Fig 5A) and total isometric tension (Fig 5B)). The presence of RGD improved the MSC-seeded scaffold contractility. Subsection 4 presented the Frank-Starling phenomenon characterized by the increase in active tension as the preload or initial length of the collagen scaffold increased (Fig 4). This major mechanical property indicates a certain degree of ultrastructural organization of both actin and non-muscle myosin filaments (NMMIIA). The RDG increased the active tension curve according to the initial length of the scaffold. Subsection 5 reported the properties of the NMMIIA at the molecular level using the Huxley formalism. The use of Huxley’s equations requires the Hill’s hyperbolic relationship between the peak shortening velocity and isotonic tension (T-V relationship). This hyperbolic T-V relationship was observed in collagen scaffolds seeded with MSCs, with or without RDG (Fig 3A, 3B and 3C). This made it possible to determine the nM concentration of CBs per g of tissue, the number of CBs per mm^3^, the maximum ATPase activity of the NMMIIA, the force of a single actin-NMMIIA CB (Fig 6), the kinetics of attachment (f1) and detachment (g1 and g2) of the actin-NMMIIA CB and the catalytic constant (kcat) (Fig 7). The RDG modified all the molecular parameters of the NMMIIA, excepted the force of the actin-NMMIIA CB and the maximum CB efficiency.

### Physical properties of scaffolds seeded with MSCs

Sixteen control scaffolds and ten RGD-decorated scaffolds were analyzed. The basic physical properties of the scaffolds laden with MSCs, i.e. the properties observed before the application of the KCl stimulus (mean diameter, CSA, weight, basal resting tone and cell counts), are presented in Table 1. None of these parameters differed statistically between scaffolds with (n = 10) and without RGD (n = 16).

**Table 1:** Morphological scaffold parameters.

	COL-MSC	COL-MSC-RGD	
**Diameter (mm)**	5.1±1.0	4.9±0.9	NS
**CSA (mm**^**2**^**)**	2.2± 0.7	2.4±0.8	NS
**Weight (mg)**	21.2±8.6	23.1± 11.8	NS
**Basal tone (mN/mm**^**2**^**)**	0.07± 0.13	0.11±0.14	NS
**Cell count**	3.6 E4 ± 6.3 E3	2.8 E4 ± 1.4 E4	NS

Mean values ± SD of scaffold diameter, cross-sectional area (CSA), weight, basal resting tone and cell counts. No significant differences were observed between COL-MSC and COL-MSC-RGD scaffolds were observed.

### Passive parameter: The Young modulus of scaffolds seeded with MSCs

The Young modulus was established for each type of scaffolds laden with MSC (Fig 2C). It averaged 1161 ± 623 Pa for RGD-functionalized scaffolds (n = 10), and 1377 ± 1220 Pa for control scaffolds (n = 16). The difference was not statistically significant (p = 0.51).

### Classical mechanical parameters of MSC control scaffolds (n = 16) and MSC-RGD scaffolds (n = 10)

The maximum rate of shortening at zero-load (Vmax) and total isometric tension (TT) was significantly greater in RGD scaffolds than in the control scaffolds (p = 0.001) (Figs 3, 5A and 5B, respectively). The percentage of maximum shortening length (max SL) was significantly higher in scaffolds with RGD than in those without RGD (0.020± 0.014 L/Lo versus 0.012±0.008 L/Lo, respectively (p = 0.022). On the other hand, the curvature G of the hyperbolic relationship between peak shortening velocity and the level of isotonic tension did not differ between the two types of scaffolds (p = 0.830) (Fig 5C).

### The Frank-Starling mechanism in scaffolds seeded with MSCs

With 0.05 M KCl, the gradual stretching of a scaffold by increments of 0.1 mm increased the isometric tension linearly (Fig 4C). The slope of the isometric tension-length relationship was significantly greater in the COL-MSC-RGD-KCl scaffolds than in the COL-MSC-KCl (p = 0.01) (Fig 4A). In the presence of BDM and KCl, the slope decreased significantly in both scaffolds with or without BDM (Fig 4A). However, the slope in scaffolds with RGD remained greater than in scaffolds without RGD (p = 0.03). The active tension (total tension minus preload) followed the same profile as the slope of the tension-length relationship, but significantly differed only in COL-MSC-KCl versus COL-MSC-KCl-BDM scaffolds (Fig 4B).

### Molecular NMMIIA-CB parameters determined by A. Huxley’s equations in scaffolds seeded with MSCs

The number of nanomoles of NMMIIA-CB per gram of tissue was 5 times greater (p = 0.0001) (Fig 6A), while the number of actin-myosin CBs per unit of volume was 6 times greater (p = 0.0001) (Fig 6B), and the NMMIIA-ATPase activity (equal to kcat x NMMIIA molar concentration) was 11 times greater in scaffolds with RGD than in those without (p = 0.0001) (Fig 6C). In contrast, the unitary NMMIIA-CB force (p = 0.709) (Fig 6D) and the maximum CB efficiency did not differ between scaffold types with and without RGD (Fig 5D). The detachment constant (g2) was significantly higher in scaffolds with RGD than in those without (p = 0.001) (Fig 7B). However, no significant differences were observed between the two types of scaffolds with respect to the detachment constant (g1) (p = 0.503) (Fig 7A) and the attachment constant (f1) (p = 0.43) (Fig 7C). Finally, the catalytic constant (kcat) did not differ between the two types of scaffolds (p = 0.349) (Fig 7D). The higher value of NMMIIA ATPase activity observed in RGD-scaffolds (Fig 6C) was due to the higher value of NMMIIA molar concentration compared to that observed in the control scaffolds (Fig 6A).

## Discussion

In this study, we devised an artificial contractile system comprising human bone marrow cells (MSCs) seeded into 3D-collagen scaffolds with and without the RGD motif. These 3D-collagen scaffolds exhibited similar contractile properties to those of the human placental tissue [14, 33, 34]. All scaffolds laden with MSCs, with or without the RGD motif, exhibited two fundamental contractile properties that are observed in all striated (cardiac and skeletal) as well as smooth muscles, namely the Frank-Starling mechanism (Fig 4) [15, 16, 35] and the Hill’s hyperbolic T-V relationship (Fig 3) [17]. The RGD motif increased the contractile level of the collagen scaffolds seeded with MSCs, as attested by the increase in maximum unloaded shortening velocity of scaffolds, and modified certain molecular contractile properties of the NMMIIA myosin, such as the nM concentration of CBs per g of tissue, the number of CBs per mm^3^, the maximum ATPase activity of the NMMIIA (Fig 6), the kinetics of attachment (f1) and detachment (g1 and g2) of the actin-NMMIIA CB and the catalytic constant (kcat) (Fig 7). The RGD motive did not modify the force of the actin-NMMIIA CB and the maximum CB efficiency. In earlier studies, we described the materials and methods to construct an artificial human contractile tissue by means of MSCs from femoral bone marrow (BM), amplified in 3D cultures and complemented with HPL [1]. BM-derived MSCs introduced in collagen scaffolds were observed to spontaneously differentiate into contractile myofibroblasts. We saw that collagen scaffolds containing myofibroblasts were able to contract after stimulation by either an electrical field or 0.05 M KCl, due to the presence of both α-SMA and NMMIIA, and without the presence of muscle myosin (MMI or MMII). We have previously shown that preparations containing myofibroblasts become contractile after stimulation by either electrical tetanus or KCl. This has been reported in the human placenta [14, 19] and in MSC-seeded collagen scaffolds [1]. This contractility is due to the presence of myofibroblasts containing NMMIIA [1, 20]. Moreover, there was no MMI and MMII as previously shown [1].

In the present study, experiments were performed within the same period and with the same biological samples used in our previous study [1]. Thus, we performed experiments with MSCs obtained from the same donors, loaded on collagen foam scaffolds which were used in [1]. We extended previous observations by assessing the number of NMMIIA-CBs per unit of volume, the NMMIIA molar concentration, the maximum NMMIIA-ATPase activity, the constants of attachment and detachment of NMMIIA-CBs, the unitary NMMIIA-CB force and the maximum NMMIIA-CB efficiency. The duration of the contractile cycle is very long (Fig 1D), as attested by the low value of the catalytic constant (kcat) (Fig 7D), which is the opposite of the duration of the CB cycle that is 5000 times higher in the heart than in MSC-laden collagen scaffolds [32]. Moreover, the duration of contraction was very prolonged under KCl (Fig 3D). In addition, we examined whether the introduction of the covalent binding of the RGD motif in collagen scaffolds was likely to modify the NMMIIA contractile performance of collagen scaffolds. The classical mechanical parameters Vmax and total tension were of the same order of magnitude as values previously observed in non-muscle human placenta [14] but were dramatically lower than those observed in the heart [32]. The heart muscle / MSC-collagen scaffold ratio for Vmax was 120. The heart muscle / MSC-collagen scaffold ratio for total tension was 170.

Surprisingly, we observed that under KCL stimulation, MSC-collagen scaffolds exhibited the Frank-Starling mechanism, i.e. the active isometric tension increased with increases in initial length [15, 16, 35]. This property is most generally observed in striated muscles (cardiac and skeletal), but it has also been observed in smooth muscles as well as a non-muscle structure, namely the human placenta [14] in which myofibroblasts represent the most numerous cells [4]. This shows that this key mechanical property is not specific to striated muscles, but is shared by both muscles and non-muscles such as the human placenta and MSC-loaded collagen scaffolds. From an ultrastructural point of view, the presence of this property strongly suggests that non-muscle structures are sufficiently organized to enable an active isometric tension increase in response to increase in initial length by increasing the degree of overlapping of the number of active CBs on NMMIIA filaments along α-SMA filaments [36]. The lowering of the slope of the isometric tension versus initial length relationship under BDM (Fig 4A), which is an inhibitor of actin-myosin CBs, clearly confirms that the initial length of the MSC-laden collagen scaffolds is a major determinant of the number of active actin-myosin CBs that interact, as observed in striated muscles.

Among the mechanical properties of the MSC-laden scaffolds, the second surprise was the observation of the hyperbolic tension-velocity relationship [17, 37]. This major mechanical property, like the Frank-Starling phenomenon, is the prerogative of most striated and smooth muscles, but also of non-muscular structures such as the human placenta [14]. By incorporating parameters of the A.V. Hill hyperbola (i.e. values of asymptotes and the G curvature of the hyperbola) into his phenomenological myosin CB theory, A. Huxley [18] has established a formalism for calculating the main molecular CB properties, namely CB attachment and detachment constants, unitary CB force, maximum CB efficiency, myosin catalytic constant (kcat), myosin ATPase activity, myosin molar concentration, CB number per g unit of tissue, and so on. In his seminal study, A. Huxley specified that this formalism could be applied to non-sarcomeric contractile tissues [18]. In MSC-laden scaffolds, most of the CB parameters determined from Huxley’s equations were of the same order of magnitude as those of human placenta, i.e., attachment (f1) and detachment (g1 and g2) constants, maximum myosin ATPase activity, and myosin molar concentration, although all these parameters were substantially higher in the heart compared to the values observed in MSC-laden collagen scaffolds and human placenta [19]. Conversely, unitary CB-force and maximum CB-efficiency were of the same order of magnitude in SMC-laden collagen scaffolds and placenta, but these two parameters were notably lower in the heart than in MSC-collagen scaffolds and human placenta [19, 32].

We showed that the functionalization of the solid collagen scaffold with the RGD motif in the presence of MSCs increased the contractile properties of preparations. All scaffolds containing MSCs decorated with the RGD motif exhibited major changes compared with control collagen scaffolds: maximum shortening velocity and total isometric tension increased in the presence of the RGD motif (Fig 5). The increase in NMMIIA-ATPase activity in the RGD scaffolds paralleled the increase in maximum shortening velocity [38]. The increase in total tension in scaffolds with RGD was mainly due to a dramatic increase in NMMIIA molar concentration (Fig 6A). They also presented the Frank-Starling mechanism. However, the slope of the isometric tension versus preload relationship was greater in scaffolds with RGD but decreased in the presence of both KCl and BDM, which inhibits the NMMIIA. This reinforces the idea that the Frank-Starling mechanism is underpinned by the overlap of NMMIIA and α-SMA filaments and that the preload determines the number of NMMIIA-CBs that are interactive with overlapping α-SMA filaments. Conversely, the unitary force of each CB (Fig 6D) and the maximum NMMIIA efficiency (Fig 5D) were not modified by the RGD. Thus, the two major effects of RGD on contractile collagen scaffolds were the drastic increases in both the NMMIIA-ATPase activity and the molecular myosin concentration (Fig 6A and 6C) without any change in cell number (Table 1).

### Non contractile passive mechanical properties of collagen scaffolds with and without RGD

The Young modulus has previously been shown to be significantly higher in scaffolds seeded with MSCs than in those without MSCs [1]. While the empty scaffold has a Young modulus below the 1 KPa threshold, MSCs seeded in scaffolds increase stiffness up to the optimum physiological level. However, in our study the introduction of the RGD motif did not modify the Young modulus of scaffolds seeded with MSCs (Fig 2). The Young modulus in MSC-seeded collagen scaffolds was in the range of what has been reported so far for myofibroblasts after TGF-β activation, that is from 1 to 3 kPa [9, 10, 12, 13]. The human platelet lysate used in our study contained TGF-β1 and favored myofibroblast differentiation. The availability of TGF-β1 is increased by the increased stiffness of the ECM [39].

### Specific contribution of the linear Arg-Gly-Asp (RGD) in the contractile function of myofibroblasts in MSC-seeded collagen scaffolds

RGD has been first described as a sequence within fibronectin that mediates cell attachment. The linear RGD is the main adhesion molecule for integrin receptors αvβ5, αvβ3 and α5β1. Interactions of integrin receptors with small adhesion peptides present on proteins of the ECM such as the collagen or fibronectin are known to mediates both cell-substratum and cell-cell interactions [40]. The functionalization of solid collagen scaffold with the RGD adhesion peptide has been shown to improve the contractile performance of preparations with neonatal cardiomyocytes [23]. In RDG collagen scaffolds, the increase in contractile performance might be partly explained through interactions with some integrins expressed by associated cells in the 3D environment. The α5β1, αvβ3, αvβ5 key integrins receptors for the linear RGD have been shown to be present on the surface of MSCs or myofibroblasts [41–44].

## Conclusion

We designed an artificial functional contractile tissue comprising human MSCs seeded in collagen scaffolds whose contractility could be improved by coupling the RGD peptide to the collagen prior to scaffold-cell seeding. The fundamental mechanical properties observed in all striated and smooth muscles were also observed in MSC-laden scaffolds, namely the Frank-Starling phenomenon and the A.V. Hill T-V hyperbolic relationship. The Frank-Starling phenomenon indicated that there was a relatively high level of ultrastructural organization of actin and NMMIIA molecules within the cells residing in collagen scaffolds, probably allowing actin filaments to slide along non-muscle myosin IIA filaments. Moreover, the chemical binding of the RGD peptide to the collagen scaffolds modified certain fundamental molecular properties of NMMIIA-CBs within the myofibroblasts. This led to an increase in contractility characterized by an increase in maximum shortening velocity and total tension. At the molecular level, the RGD motif increased the kinetics of detachment of NMMIIA-CBs as well as the molar NMMIIA concentration and the maximum NMMIIA-ATPase activity without altering the unitary force of NMMIIA-CBs and their maximum efficiency. Thus the artificial tissue we designed developed exceptional contractile properties that can be compared with those previously observed in vivo in other non-muscle contractile tissues such as the human placenta.
